# A DO- and pH-Based Early Warning System of Nitrification Inhibition for Biological Nitrogen Removal Processes

**DOI:** 10.3390/s121216334

**Published:** 2012-11-26

**Authors:** Seil Hong, Il Choi, Byung Jin Lim, Hyunook Kim

**Affiliations:** 1 Department of Environmental Engineering, University of Seoul, 90 Jeonnong-dong Dongdaemun-Gu, Seoul 130-743, Korea; E-Mails: seilhong@uos.ac.kr (S.H.); il_choi@hangulmail.kr (I.C.); 2 National Institute of Environmental Science, Incheon 404-708, Korea; E-Mail: limnolim@korea.com

**Keywords:** nitrification inhibition, pH, DO, early warning system, biological nitrogen removal processes

## Abstract

In Korea, more than 80% of municipal wastewater treatment plants (WWTPs) with capacities of 500 m^3^·d^−1^ or more are capable of removing nitrogen from wastewater through biological nitrification and denitrification processes. Normally, these biological processes show excellent performance, but if a toxic chemical is present in the influent to a WWTP, the biological processes (especially, the nitrification process) may be affected and fail to function normally; nitrifying bacteria are known very vulnerable to toxic substances. Then, the toxic compound as well as the nitrogen in wastewater may be discharged into a receiving water body without any proper treatment. Moreover, it may take significant time for the process to return back its normal state. In this study, a DO- and pH-based strategy to identify potential nitrification inhibition was developed to detect early the inflow of toxic compounds to a biological nitrogen removal process. This strategy utilizes significant changes observed in the oxygen uptake rate and the pH profiles of the mixed liquor when the activity of nitrifying bacteria is inhibited. Using the strategy, the toxicity from test wastewater with 2.5 mg·L^−1^ Hg^2+^, 0.5 mg·L^−1^ allythiourea, or 0.25 mg·L^−1^ chloroform could be successfully detected.

## 1. Introduction

In order to protect national waters from unwanted algae blooming, stringent regulations on the water quality of the effluents from wastewater treatment plants (WWTPs) has been imposed in Korea; especially, the regulation on the nitrogen levels is getting stricter. In practice there is no realistic way to purify huge amounts of wastewater without the aid of microorganisms (*i.e.*, activated sludge). In fact, all 470 WWTPs with a capacity of 500 m^3^·d^−1^ or more in Korea utilize the activated sludge process for treating wastewater [1].

In urban areas, the joint treatment of industrial wastewater and domestic wastewater is commonly practiced [2]. Therefore, there exists a potential risk that toxic substances, including heavy metals, organic compounds, and nanoparticles could be released from an industry to a WWTP via various routes, e.g., intentional or unintentional spills, and leaking pipes [3]. Also, during a storm, a number of toxic chemicals on the surface of urban areas can flow into a WWTP via storm runoff, especially in a combined sewer service area; about 87% of Metropolitan Seoul is provided with combined sewer service [4]. Elsewhere, it has been reported that 45–60% of Swedish WWTPs were found to receive wastewater containing inhibitory substances [5].

In a nitrogen removal process, the nitrification step in which ammonia in wastewater is oxidized to nitrate by autotrophic nitrifiers is essential, since it is the perquisite to denitrification in which nitrate produced from nitrification is reduced to N_2_ gas, which is ultimately removed from water. These nitrifying bacteria are characterized by two distinct properties: slow growth rate and vulnerability to toxic compounds [5,6]. Therefore, if the nitrifying bacteria are exposed to toxic compounds, they easily lose their ability to oxidize ammonia in water. Moreover, it takes considerable time for the autotrophic nitrifying bacteria to return to their normal state, comparing to heterotrophic organic-oxidizing bacteria [7].

Therefore, a screening method for influent wastewater to identify toxicants early should be prepared to take a preventive action for minimizing adverse effects caused by the toxicants on the main process (*i.e.*, activated sludge process) in a WWTP. A number of technologies have been proposed to identify upsets of nitrifying bacteria by toxins in a WWTP; for examples, respirometry [7–9], titrimetry [10], off-gas measurement [11,12], and bioluminescence [13]. Some of the technologies are based on a bioreactor with specific microbial species and analyze the result of the microbial reaction with wastewater fed into the bioreactor. These technologies often require a very extensive calibration step but are still very sensitive to operational and environmental conditions such as water temperature, pH, and the amount of nitrogen in wastewater; because they are very dependent on the absolute values presented by sensors. Therefore, sometimes they function abnormally since the characteristics of the wastewater sent to a WWTP are often very complex and variable. In order to more appropriately screen wastewater containing nitrification-inhibiting substances, a microbial sensing system utilizing a mixed culture (e.g., activated sludge) with nitrifying bacteria would be better than the one using a pure culture [14]. In addition, a strategy to identify any reduction in the activity of the nitrifying bacteria without relying on absolute values of signals from sensors should be developed.

Under normal conditions, nitrifiers consume more oxygen to oxidize NH_4_^+^ to NO_2_^−^ or NO_3_^−^ than heterotrophs do to oxidize organics to CO_2_; thus, 4.2 mg oxygen is required for oxidizing 1 mg NH_4_^+^, while about 0.5 mg oxygen is consumed for oxidizing 1 mg organics [15]. In addition, the nitrification rate is so fast that the oxygen consumption rate over time (*i.e.*, dDO/dt) is also fast. Therefore, for a given wastewater, dDO/dt of activated sludge with nitrifying bacteria is steeper than the one without nitrifying bacteria. In fact, the oxygen consumption rate (also called oxygen uptake rate (OUR)) has been utilized for directly assessing the activity of nitrifying activated sludge [16]. Once the activity of nitrifying bacteria is inhibited, and it leads to decreased oxygen consumption rate in the system [17]. In fact, several technologies such as the NITRification tOXicity tester (NITROX, [18]) and selective nitrification inhibitor addition respirometry [19] have been applied to detect nitrification inhibition. Since these methods distinguish the oxygen uptake by nitrifying bacteria from that by total sludge, a nitrification inhibitor should be added. Therefore, these technologies require extra equipment to inject a nitrification inhibitor into test wastewaters.

Practically speaking, oxygen consumption by nitrification processes accounts for approximately 40% of the total oxygen demand in an advanced WWTP [20]. Reduced nitrifier activity will clearly affect the oxygen consumption rate of the total activated sludge, therefore, if the activated sludge is enriched with nitrifying bacteria [21]. This means that distinction between the oxygen uptake by the nitrifiers and the total activated sludge one may not be necessary to screen wastewater for potential toxicity.

**Figure 1.**
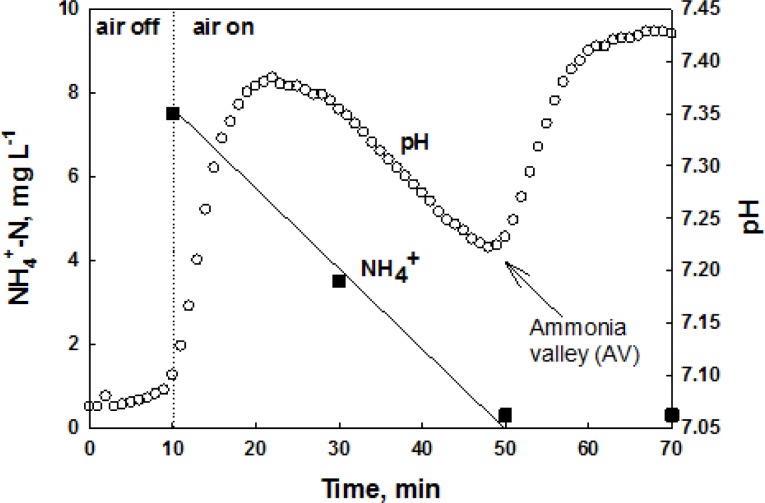
pH and NH_4_^+^ profiles during nitrification.

The nitrification process consumes system alkalinity. Therefore, the system pH can be lowered during nitrification of wastewater ammonia. However, if the nitrification is terminated, the system pH can be slightly increased due to the ammonification of organic nitrogen, resulting in a distinct inflection point (*i.e.*, dpH/dt = 0) on the pH profile of the system; this inflection point has been called “ammonia valley (AV)” [22]. For readers’ better understanding, the pH profile of an intermittently aerating activated sludge process for nitrogen removal during aerobic cycle is provided along with NH_4_^+^ concentration in Figure 1. In this figure, when the oxidation of NH_4_^+^ is completed a valley can be drawn on the pH profile. If nitrifiers are inhibited, the occurrence of the AV on the pH profile will be delayed or will not be detected at all.

In fact, the AV has been utilized by others to control the aeration of intermittently aerating activated sludge systems [23]. However, the inflection point on the pH profile has not been utilized for screening wastewater to early identify the present of toxicity-causing substances. In this study, therefore, a DO- and pH-based strategy to identify potential nitrification inhibition was developed to detect early the inflow of toxic compounds to a biological nitrogen removal process, before the process completely fails. Using the data collected from a reactor equipped with a DO probe during the nitrification, dDO/dt was calculated, while using the ones from the other reactor, the time for the AV (*i.e.*, dpH/dt = 0) to occur on the pH profile was calculated. To simplify calculation of the respiration rate of the activated sludge, the respirometic measurement was carried out under static gas conditions, which is referred to as a closed respirometer [20,24]. The feasibility of the strategy was first evaluated by performing experiments in a batch mode with toxic chemicals. Then, a sequencing batch reactor type toxicity detector was built to continuously screen wastewater for the presence of potential inhibition-causing substances.

In this study, three chemicals (*i.e.*, Hg^2+^, allythiourea (ATU), and CHCl_3_) were used as toxicity- causing substances. Urban storm water runoff may contain heavy metals, solvents, hydrocarbons, and other toxic chemicals [26]. Sources of heavy metals in runoff could be automobiles or industrial plants. Heavy metals could be detected up to ppm levels in urban storm water [27]. It is true that the detection of mercury at high concentration from storm water runoffs is not common [28], but it might be detected in runoff from areas where the metal finishing industries are located [29]. Furthermore, since mercury is considered as one of the most toxic heavy metals to microorganisms [30], exposure of activated sludge to the metal should be avoided. Therefore, mercury was selected as a test toxicant in this study. Since allythiourea is typically used to prevent nitrification from occurring in a BOD_5_ test [25], it was selected as a test toxicant in this study. CHCl_3_ also has been known as a nitrification inhibitor [31], therefore, it was selected along with other toxicants in this study.

Since the strategy proposed in this study to screen wastewater for potential toxicity to nitrifiers is based on the dDO/dt and dpH/dt profiles obtained during nitrification, it is not affected much by the shift in absolute values obtained from each sensor.

## 2. Material and Methods

### 2.1. Activated Sludge and Wastewater

The activated sludge used in this study was procured from the aeration basin of a pilot scale aerobic-anoxic-oxic system of 50 m^3^·d^−1^ operated in a local advanced WWTP. Once it was collected, the activated sludge was transported to the laboratory. Then, moisturized air was supplied to the activated sludge for more than 3 h in order to oxidize any remaining organics and ammonia. After the pretreatment, the activated sludge was transferred to two 4 L batch reactors. A DO probe was installed on the one of the reactors and a pH probe was installed on the other. The temperature of the reactors was carefully maintained at 25 ± 1 °C since the activity of nitrifying bacteria is affected by changes in temperature.

The effluent from a primary sedimentation basin of the pilot plant was used as test wastewater, to which a toxicant was added. The general characteristics of the wastewater (*i.e.*, COD, TKN, NH_4_^+^, NO_3_^−^, and alkalinity) and activated sludge (*i.e.*, mixed liquor volatile suspended solids: MLVSS) are summarized in Table 1. All the parameters were analyzed according to the Standard Methods [25].

### 2.2. Toxicants Applied in This Study

To evaluate the feasibility of the proposed strategy, three toxic chemicals were arbitrarily selected. They are Hg^2+^ (as HgCl_2_), ATU (C_4_H_8_N_2_S), and chloroform (CHCl_3_). The selected test toxicants of analytical grade were purchased from Sigma-Aldrich (St. Louis, MO, USA). Firstly, a stock solution of each selected chemical was made and stored under −20 °C until used. Whenever needed, the stock solution was diluted to an appropriate concentration with deionized water. Table 2 shows the concentration levels of each toxicant applied in this study along with published concentrations causing inhibition to activated sludge.

### 2.3. Data acquisition and Filtering

Changes in the OUR and in the pH profiles of the reactors with nitrifying activated sludge were monitored using a DO probe (DO-350L, i-stek, Incheon, Korea) and a pH probe (HA 405-DXK-S8, Mettler Toledo, Zurich, Switzerland). Both sensors were connected to a data acquisition and control (DAC) module (NI cDAQ-9172, National Instrument, Austin, TX, USA). The visualization of signals from the sensors and the DAC module, and the nitrification inhibition detection were made using a graphic user interface development environment (LabVIEW 8.2; National Instruments).

Normally, the signals from the DO and the pH probes contain concomitant noises. Therefore, it is hard to draw their true profiles. Thereafter, a moving-average digital filter was implemented for smoothing data from sensors (Equation (1)). The pH and DO of reactors were collected 10 times a second, and they were averaged every 20 s; 200 data (N*) were averaged to produce one data point. Then, the derivatives of the pH and DO (*i.e.*, dpH/dt and dDO/dt) were calculated once every 1 min [22], since the biological reaction did not change spontaneously.

(1) $$y_{F}\left(k\right)=\frac{1}{N^{*}}\sum_{i=k-N^{*}+1}^{k}y_{i}$$

where N* is the number of past data points that are being averaged.

### 2.4. Batch Assay Experiments

In the beginning, 3 L of nitrifying activated sludge was filled into each of two 5 L reactors (working volume: 4 L). Then, 1 L wastewater was added to each reactor. Once wastewater was added, the aerator for each reactor was turned on to provide air. In the case of the reactor with the DO probe installed, the reactor DO was initially increased up to 7 mg·L^−1^. Then, the aerator was turned off to monitor the DO profile and calculate dDO/dt (or OUR); during the air-off period, the liquid of the reactor was slowly mixed using a magnetic stirrer for keeping the sludge in suspension. If the reactor DO was lower than 3 mg·L^−1^, then the aerator was turned on again to increase the DO to 7 mg·L^−1^. While the reactor DO was raised to 7.0 mg·L^−1^, the OUR was not calculated. In the case of the reactor with the pH probe installed, the reactor pH was monitored to calculate dpH/dt and to identify the inflection point (*i.e.*, AV or dpH/dt = 0) on the pH profile during the aeration.

During each batch assay experiment, a 30 mL mixed liquor sample was collected once every 20 min directly from the reactor with the pH probe installed. Then, NH_4_^+^ concentration of the samples was analyzed to demonstrate the relation between the pH profile and NH_4_^+^ oxidation by nitrifiers during the air-on time [22].

### 2.5. Detection of Nitrification Inhibition in Sequencing Batch Reactor Type Detector

**Figure 2.**
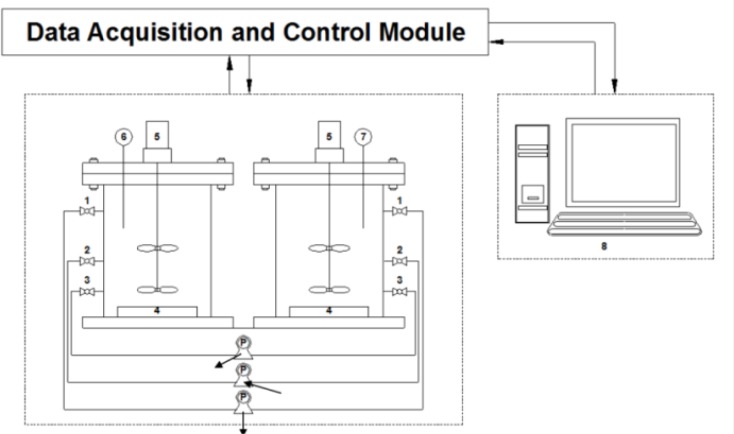
Schematic diagram of toxicity detector used in this study. (1) decanting line; (2) feeding line; (3) sludge wasting pump; (4) air diffuser; (5) mixer; (6) DO probe; (7) pH probe; (8) personal computer.

After the batch bioassay experiments, a toxicity detector was built in order to continuously screen wastewater for the presence of a potential toxicant (Figure 2). The toxicity detector consisted of two reactors with a working volume of 3 L each, peristaltic pumps, air diffusers, magnetic stirrers, a pH probe, a DO probe, a DAC module, and an IBM-compatible PC. Feeding and draining test wastewater, turning air on/off, turning a mixer on/off, collecting signals from the DO and pH sensors and calculating the derivatives of the DO and pH profiles for detecting the presence of toxicants were realized with the DAC module and a program coded with LabVIEW 8.2.

**Figure 3.**
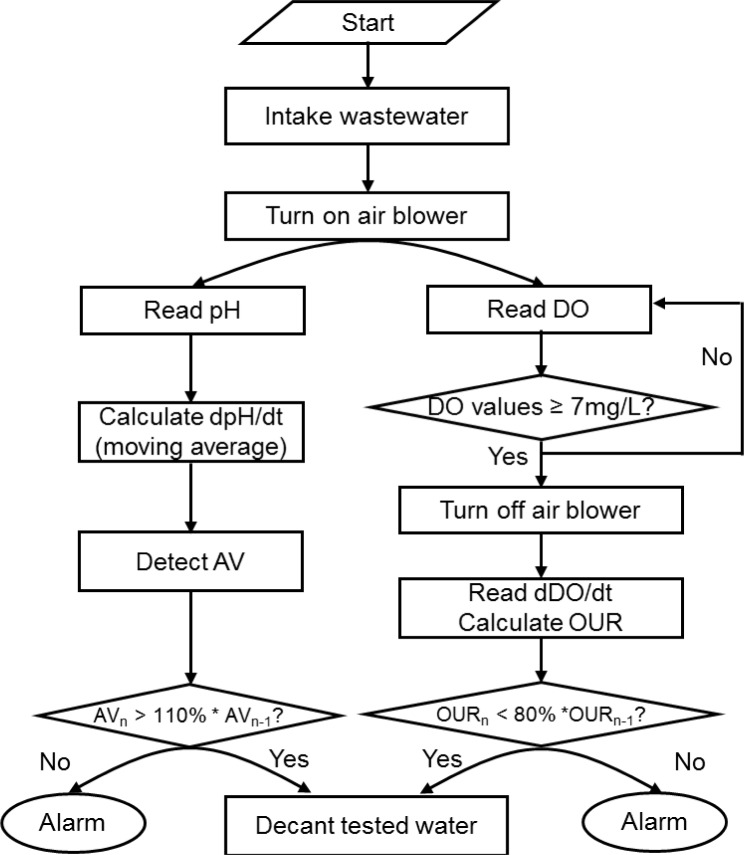
Proposed logic flow to detect toxicity (AV_n_: AV detection time in current cycle, AV_n-1_: AV detection time in previous cycle).

Each of the two reactors was filled with 2.7 L activated sludge, which had been procured from the same pilot plant mentioned above. As in the case of the batch assay, changes on the pH and DO profiles drawn during nitrification were monitored to detect the inhibition to the activity of nitrifiers. A program logic for screening wastewater to identify the presence of potential toxicants is presented in Figure 3.

In case of the reactor with a pH probe, initially 300 mL wastewater (10% of the total reactor working volume) was added. The amount of sample provided to the toxicity detector was minimized to reduce the NH_4_^+^ concentration in the reactor and to more rapidly detect the AV on the pH profile. Once the feeding of wastewater was completed, air was supplied to maintain the reactor DO at 4.0 mg·L^−1^. Then, the reactor pH was monitored to calculate dpH/dt for identifying the AV on the pH profile (*i.e.*, the termination of nitrification). Once the AV was detected, the activated sludge was allowed to settle down and the supernatant was removed from the top of the reactor. The new wastewater was then added to the reactor for the next run (Figure 3). If the time elapsed to detect the AV in the current measurement was 10% longer than the one of the previous measurement, the PC issued a warning.

In case of the reactor with a DO probe, the oxygen uptake by the activated sludge was monitored to calculate the OUR. If the calculated OUR was 20% or more lower than the one determined in the previous measurement, the PC issued a warning for the inflow of potential toxicants.

## 3. Results and Discussion

### 3.1. Bioassay Tests in Batch Mode

**Figure 4.**
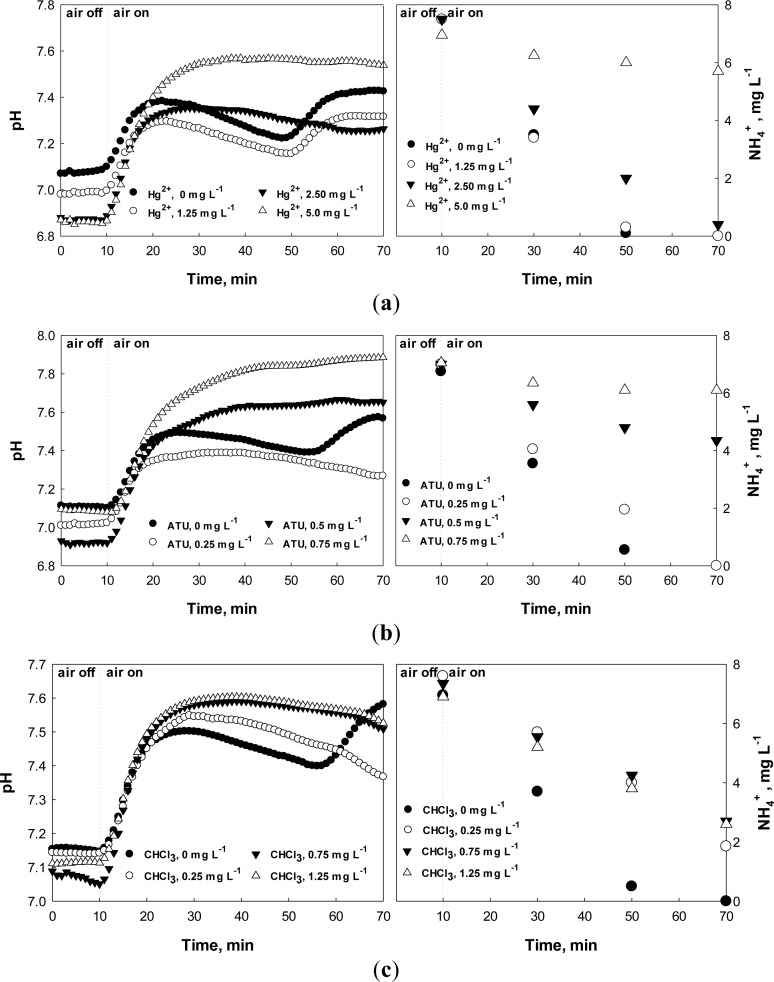
pH and NH_4_^+^ profiles during nitrification in presence of (**a**) Hg^2+^, (**b**) ATU, and (**c**) CHCl_3_. Air turned on at time 10 min.

Batch assay tests were performed and the effects of the toxicants (*i.e.*, Hg^2+^, ATU, and CH_3_Cl) on the nitrification process were evaluated by monitoring the pH and DO profiles. During the nitrification, in general, the system pH first decreases due to the H^+^ from nitrification process. However, if the nitrification is completed, the pH increases again via ammonification, producing a local minimum (*i.e.*, AV) on the pH profile (Figure 4(a–c)) [22].

Under normal conditions, the AV on the pH profile could be identified easily within less than an hour. However, if the activity of nitrifiers was negatively affected, the time for the AV detection could be prolonged. Figure 4 demonstrates that the ending time of nitrification and the time elapsed to detect the AV on the pH profile are identical. In this specific experiment, the nitrification was complete within 40–50 min after the aeration was initiated; the completion of the nitrification is characterized by the AV on the pH profile. In fact, the time for the AV occurrence on the pH profile was inversely proportional to the nitrification rate, and was proportional to the amount of a toxic chemical added. In other words, the time would be proportional to the degree of the nitrification inhibition, which is calculated using Equation (2):

(2) $$\mathrm{Percent}\;\mathrm{nitrification}\;\mathrm{inhibition}\left(\%\right)=\frac{{\mathrm{AUR}}_{\mathrm{normal}}-{\mathrm{AUR}}_{\mathrm{with}\;\mathrm{toxicant}}}{{\mathrm{AUR}}_{\mathrm{normal}}}\times 100\%$$

where, *AUR_normal_* and *AUR_with toxicant_* are the ammonia utilization rate (mg NH_4_^+^ L^−1^·min^−1^) under the normal condition and in the presence of a toxicant, respectively.

When 5 mg·L^−1^ Hg^2+^ was present in the test wastewater (Figure 4(a)), the AV could not be observed on the pH profile, indicating very slow (or no) nitrification had occurred. In fact, based on the calculation with data provided in Figure 4(a), more than 70% nitrification inhibition was observed. ATU and CHCl_3_ caused significant inhibition to the activity of nitrifiers even at lower concentrations; ATU of 0.5 mg·L^−1^ or more and CHCl_3_ of 0.25 mg·L^−1^ or more could induce almost complete nitrification inhibition (Figure 4(b,c)). In the case of CHCl_3_, the nitrification was inhibited even at 0.25 mg·L^−1^ CHCl_3_. In fact, CHCl_3_ concentration of as low as 0.1 mg L^−1^ could inhibit the activity of nitrifying activated sludge (data not shown).

From Figure 4(b), when 2.5 mg·L^−1^ Hg^2+^ or 0.25 mg·L^−1^ ATU was present in the test wastewater, the AV occurrence on the pH profile was delayed for about 20 min. Since the activity of the activated sludge does not change within several hours under the normal operating condition, significantly delayed nitrification can be regarded abnormal. Therefore, the upper limit for the time delay of the AV occurrence was arbitrarily set at 10%. This means that wastewater flowing into the WWTP of interest may inhibit the activity of the activated sludge if the time for detecting the AV in the current measurement is delayed 10% or more than the previous measurement.

The percent nitrification inhibition was also assessed empirically using the changes in the OUR measurements as shown in Equation (3). In this specific experiment, the OUR value was calculated to be 0.095–0.1 mg O_2_ min^−1^ under normal conditions. At 2.5 mg·L^−1^ Hg^2+^ or 0.25 mg·L^−1^ ATU, however, the OUR of the activated sludge in the batch reactor was lowered more than 20% (Table 3); the calculated OUR was lower than 0.08 mg O_2_ min^−1^. Therefore, measuring the OUR of the total activated sludge also could play an auxiliary role in the detection of the nitrification inhibition. In fact, 20% respiration inhibition has been suggested as a guideline for deciding if test wastewater is “safe” or “toxic” to activated sludge in literature [15]:

(3) $$\mathrm{Percent}\;\mathrm{respiration}\;\mathrm{inhibition}\left(\%\right)=\frac{{\mathrm{OUR}}_{\mathrm{normal}}-{\mathrm{OUR}}_{\mathrm{with}\;\mathrm{toxicant}}}{{\mathrm{OUR}}_{\mathrm{with}\;\mathrm{toxicant}}}\times 100\%$$

where, *OUR_normal_* and *OUR_with toxicant_* are the oxygen utilization rate (mg O_2_ L^−1^·min^−1^) under the normal condition, and in the presence of a toxicant, respectively.

### 3.2. Detection of Nitification Inhibition Using OUR and AV on pH Profile

**Figure 5.**
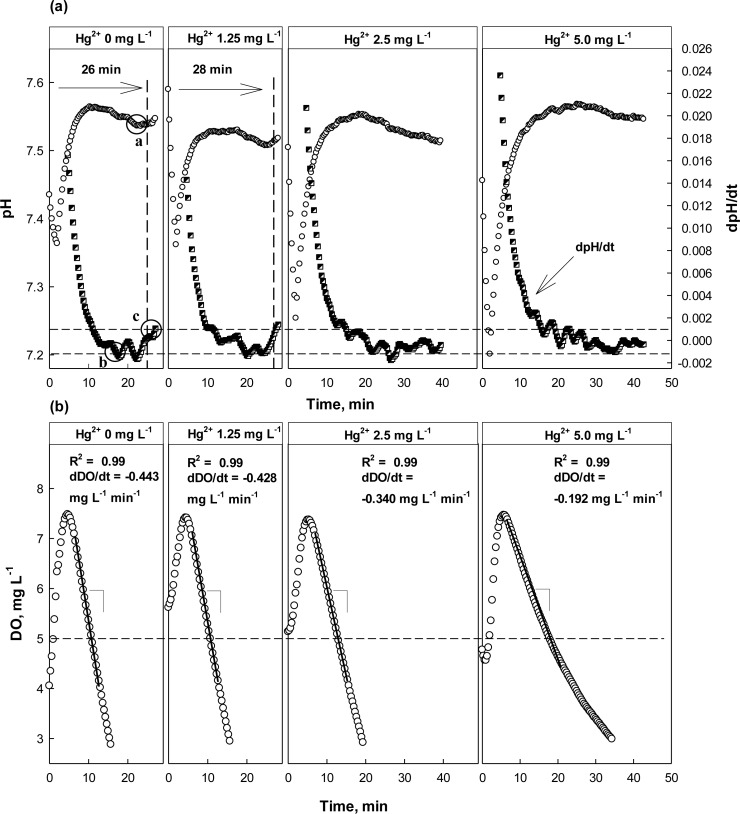
pH, dpH/dt, DO, and dDO/dt profiles with and without Hg^2+^ at different concentrations. Dashed lines: guideline set for dpH/dt to identify AV on pH profile.

**Figure 6.**
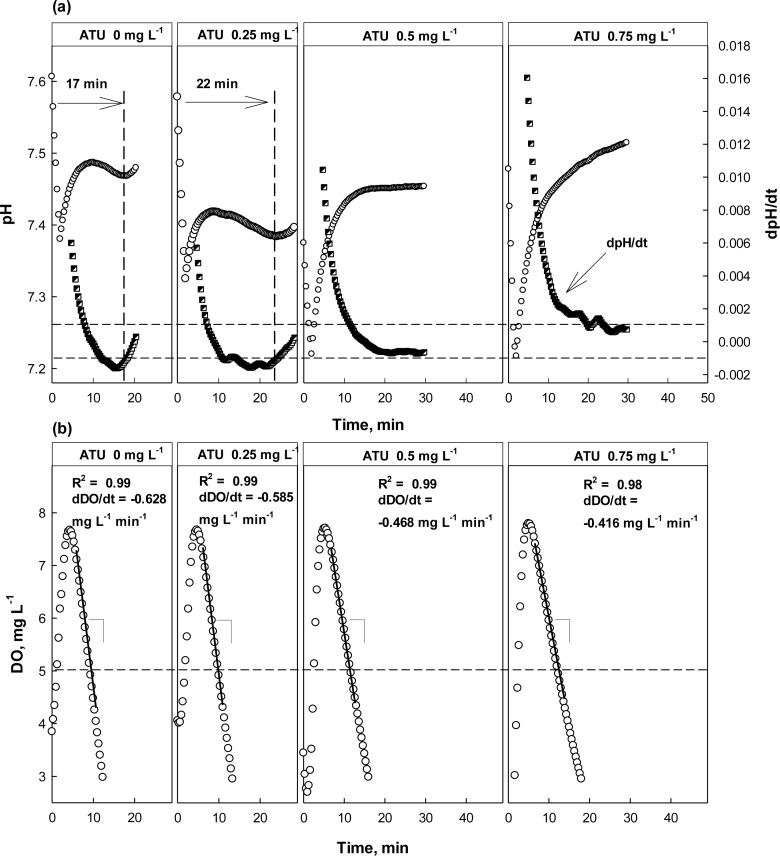
pH, dpH/dt, DO, and dDO/dt profiles with and without ATU at different concentrations. Dashed lines: guideline set for dpH/dt to identify AV on pH profile.

**Figure 7.**
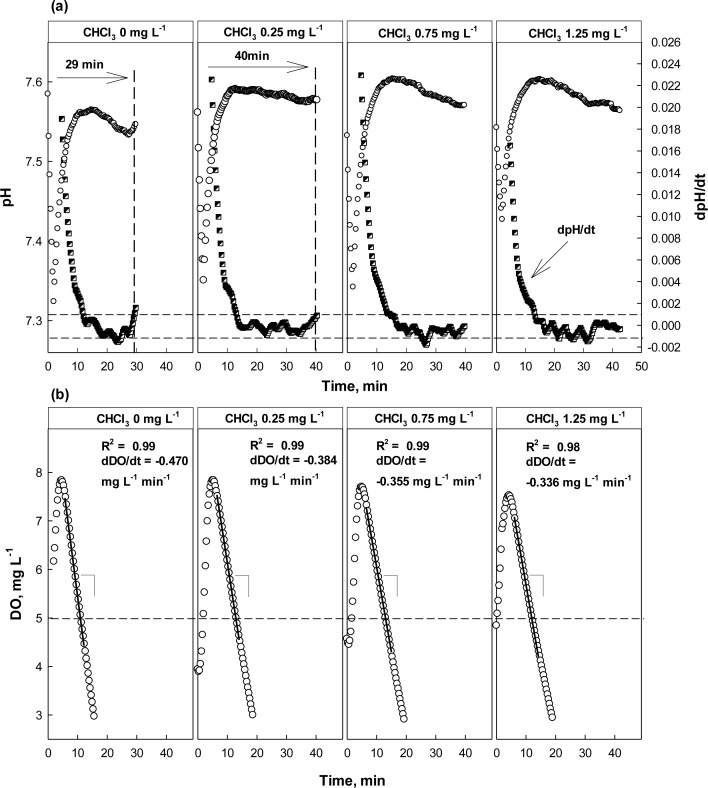
pH, dpH/dt, DO, and dDO/dt profiles with and without CHCl_3_ at different concentrations. Dashed lines: guideline set for dpH/dt to identify AV on pH profile.

As discussed above, both dpH/dt and dDO/dt of the batch reactors were monitored along with pH and DO in the presence or absence of Hg^2+^, ATU, or CHCl_3_ (Figures 5–7). When no toxic chemical was present in the test wastewater, the dpH/dt of the reactor changes from (−) to (+) at the AV, although some noises were observed. In order to reliably identify the AV on the pH profile, the strategy suggested in a previous study [31] was adopted. Namely, the AV (A in Figure 4(a)) on the pH profile was identified by detecting B (dpH/dt = −0.001) and C (dpH/dt = 0.001) on the dpH/dt profile in sequence.

The OUR (or dDO/dt) of the activated sludge was determined by performing a linear regression; the linear regression was carried out using the data collected when DO concentration was between 5 and 7 mg·L^−1^. Therefore, if the slope of the linear regression curve for the DO consumption in the current run was 20% or more less steeper than the one measured in the previous run, it was considered that the test wastewater contained toxicity-causing substances.

The feasibility of the proposed strategy was evaluated by applying it to screen wastewater with different concentrations of Hg^2+^. As shown in Figure 5(a), the inflection point (=AV) could be detected on the pH profile at 25 min after the aeration was initiated for the wastewater with 0 mg·L^−1^ Hg^2+^. However, it could be detected at time 35 min for the wastewater with 2.5 mg·L^−1^ Hg^2+^. Interestingly, the proposed method could not distinguish the toxicant-free wastewater and the wastewater with 1.25 mg·L^−1^ Hg^2+^. No distinct delay was observed in the AV detection time for the wastewater with Hg^2+^ (28 min), comparing the test with the Hg^2+^-free wastewater (26 min). The dDO/dt in the absence of Hg^2+^ was −0.443 mg·L^−1^·min^−1^, while that in the presence of Hg^2+^ was −0.428 mg·L^−1^·min^−1^ (Figure 5). It indicates that the activity of the nitrifiers was not significantly inhibited by 1.25 mg L^−1^ Hg^2+.^ In fact, it has been reported that Gram negative bacteria such as nitrifying bacteria could reduce Hg^2+^ to Hg(0) to some extent with NADPH inside the cell and could thus detoxify it [29,30,32].

However, in the presence of 2.5 or 5 mg·L^−1^ Hg^2+^ in the test wastewater, the AV on the pH profile was detected 5 min or more later than under normal conditions. As shown in the Figure 5(b), dDO/dt also indicated the nitrification being inhibited when the test wastewater contained Hg^2+^ of 2.5 mg·L^−1^ or more, showing its value of −0.340 mg·L^−1^·min^−1^ or higher.

In the case where ATU or CH_3_Cl was present in the test wastewater, similar trends could be detected in the pH and DO profiles (Figure 6). However, the result from the test with ATU clearly showed the detection of nitrification inhibition based on the dpH/dt would be superior than the one based on the OUR. Even at 0.25 mg·L^−1^ ATU, the occurrence of the AV on the pH profile delayed for about 5 min, comparing to the one at 0 mg·L^−1^ ATU; a warning was issued by the system. However, the DO profile drawn in the presence of 0.25 mg·L^−1^ ATU was not much different from the one in the absence of ATU (Figure 6(b)). If the toxicity detector system was operated only depending on the DO profile, a warning would not be issued.

At higher concentrations (*i.e.*, 0.5 and 0.75 mg·L^−1^), both the dpH/dt and dDO/dt profiles indicated the activity of the nitrifiers was being inhibited; in fact the AV could not be observed in the presence of ATU of 0.5 mg·L^−1^ or more. On the other hand, the dDO/dt for the test wastewater was also increased to reveal the nitrification inhibition.

The result of the test with CHCl_3_ as a toxicant is shown in Figure 7. Apparently, the activity of nitrifiers was seriously inhibited by the presence of CHCl_3_. In the presence of 0.25 mg·L^−1^ CHCl_3_, the occurrence of the AV on the pH profile was delayed for more than 10 min, indicating that the activity of nitrifiers inhibited. However, the DO profiles did not show significant difference between with and without 0.25 mg·L^−1^ CHCl_3_. Increase of dDO/dt which was smaller than the alarming criterion for the OUR was observed; 18% of OUR change was observed.

At higher concentrations (*i.e.*, 0.75 and 1.25 mg·L^−1^), both the dpH/dt and dDO/dt profiles indicated the activity of the nitrifiers was being inhibited; in fact the AV could not be observed in the presence of CHCl_3_ of 0.75 mg·L^−1^ or more. On the other hand, the dDO/dt for the test wastewater with 0.75 mg·L^−1^ or more was almost similar to that of wastewater with 0.25 mg·L^−1^. This means that the activity of nitrifiers could be severely inhibited by CHCl_3_ at a very low concentration. In addition, the oxygen consumption shown in Figure 6(b) might only represent the respiration by the heterotrophs in the detector system.

The result from the tests with three different toxicants at three levels is summarized in Table 4. In general, the strategy based both on the OUR and pH profiles could successfully screen test wastewater for potential inhibition to the nitrification process, except the wastewater containing 1.25 mg·L^−1^ Hg^2+^.

### 3.3. Detection of Toxicity in Continuous Feeding Mode

Based on the result from the batch bioassay experiments, a nitrification inhibition detector was built to continuously screen incoming wastewater by real-time calculating dpH/dt and dDO/dt (*i.e.*, OUR). Once new wastewater was fed into the two reactors of the detector, the detector evaluates if the wastewater contained toxic substances inhibiting the activity of nitrifiers both by checking the time taken for the AV to occur and by calculating the OUR.

If the time for the AV occurrence (*i.e.*, dpH/dt = 0) on the pH profile in the current run was 10% or more longer than the previous run, the detector was programmed to issue a warning. For the first 5 min when new wastewater was fed into the pre-tester, the reactor dpH/dt was not monitored.

In addition, if the OUR for the current run was 20% or much smaller than in the previous run, the detector was programed to issue a warning. Therefore, a warning signal could be issued if new wastewater for the current run could not pass either the screening criteria based on the dpH/dt or the one based on the OUR.

**Figure 8.**
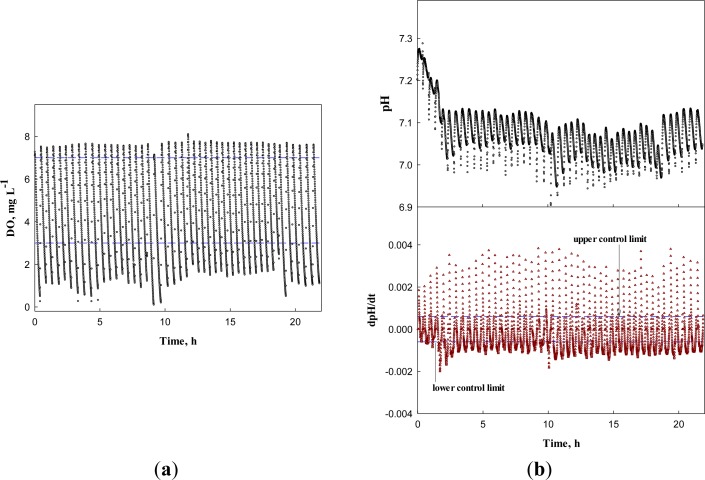
(**a**) DO and dDO/dt, (**b**) pH and dpH/dt profiles under normal condition.

First, the system was operated for the wastewater without any toxicant to obtain the DO, dDO/dt, pH, and dpH/dt profiles (Figure 8). In Figure 8(a,b), the DO and pH profiles show little change over time, except the pH decrease in the first four runs. Nonetheless, the dDO/dt and dpH/dt did not issue a warning, indicating the activated sludge in the detector system was not inhibited. Over more than 20 h, the AVs were successfully detected by the system; AVs occurred in the range between 6.95 and 7.25 with the mean of 7.03 and standard deviation of 0.06.

**Figure 9.**
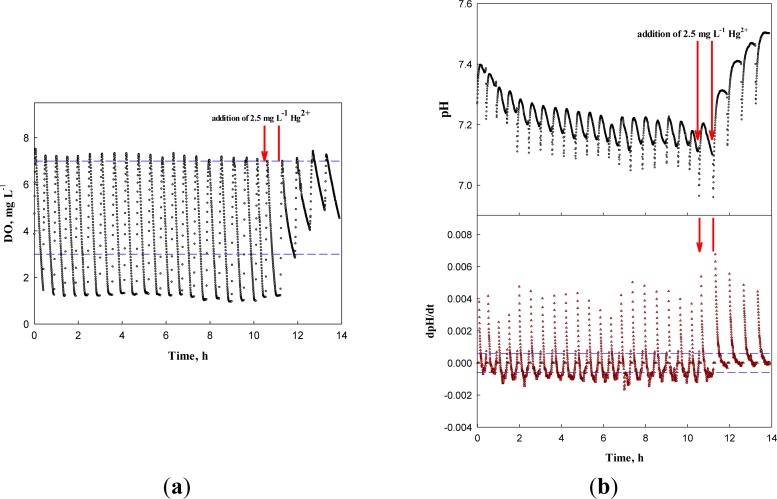
(**a**) DO and dDO/dt, (**b**) pH and dpH/dt profiles fed with wastewater containing 2.5 mg·L^−1^ Hg^2+^.

However, if test wastewater contained toxicity-causing substances, the occurrence of the AV on the pH profile was delayed. For example, when 2.5 mg·L^−1^ Hg^2+^ was present in the test wastewater, the system could easily identify the inhibition of nitrifiers with both the delayed occurrence of the AV on the pH profile and the reduced OUR (Figure 9).

As shown in the last section, if a toxicant exerting low acute toxicity to nitrifying activated sludge is present in the influent wastewater to a WWTP, the system may not screen the wastewater. In fact, 1.25 mg·L^−1^ Hg^2+^ or 0.25 mg·L^−1^ ATU did not inhibit the activity of nitrifiers much; only little delay of the AV occurrence on the pH profile could be observed. However, if wastewater containing a low concentration of chemicals continuously flows into a WWTP, it could cause a chronic toxicity in its biological processes.

**Figure 10.**
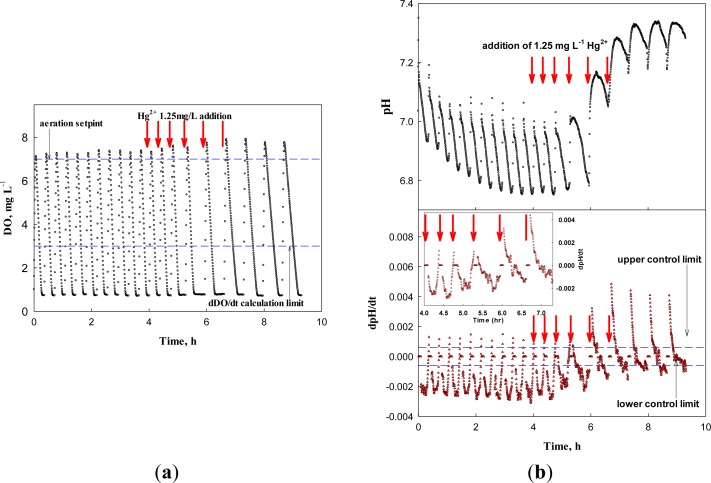
(**a**) DO and dDO/dt, (**b**) pH and dpH/dt profiles for wastewater containing 1.25 mg·L^−1^ Hg^2+^.

Therefore, the system was applied to screen the wastewater containing a low concentration of toxicant. After each test for the nitrification inhibition, the system discharged the test wastewater and was replenished with new wastewater for the next test. Figure 10 represents the system response to the continuous inflow of the wastewater containing 1.25 mg·L^−1^ Hg^2+^. For the first three cycles, the system could not detect the presence of a toxicant in the influent. However, from the 4th cycle, the relative chronic effect of Hg^2+^ of 1.25 mg·L^−1^ on the nitrifying bacteria delayed the occurrence of the AV on the pH profile significantly enough for the system to issue an warning. In fact, even with Hg^2+^ of such a low concentration, the activity of the nitrifiers was affected; the time of the AV occurrence was delayed in each run of the test (+0.5, +1.3, +7.7, and +10 min for 1st through 4th run, respectively). On the other hand, a significant change in dDO/dt could be observed in the 5th test.

## 4. Conclusions

In this paper, a DO- and pH-based strategy to identify nitrification inhibition has been implemented in a laboratory detector system, which has been applied to screen the wastewater containing either Hg^2+^ (concentration range: 0–5 mg·L^−1^), ATU (concentration range: 0–0.75 mg·L^−1^) or CHCl_3_ (concentration range: 0–1.25 mg·L^−1^). In general, the strategy for detecting the nitrification inhibition based on the AV occurrence time was superior to the one based on the OUR change. Nonetheless, in this study, the toxicity detection strategy was programmed to screen wastewater with toxicity-causing substances using both parameters.

In batch experiments with the three toxicants, the strategy proposed in this study could identify the acute toxicity caused by each toxicant at different levels, except the one caused by 1.25 mg·L^−1^ Hg^2+^; it was considered that 1.25 mg·L^−1^ Hg^2+^ might cause low acute toxicity to nitrifying microorganisms. However, a toxicity detector which was implemented with the proposed strategy and was operated in a continuous feeding mode could identify the toxicity caused by Hg^2+^ of 1.25 mg·L^−1^ within one and an half hours. Therefore, it was concluded that the strategy could screen wastewater containing toxicants inducing low acute toxicity to a biological nitrogen removal process.

In practice, this detector can be mounted at the outlet of a primary settling tank for screening wastewater to early detect toxicity from chemicals flowing into aeration basins or a biological process. If the system detects any potential toxicity by the incoming wastewater, a WWTP operator can divert the wastewater into a reservoir, and prevent the complete failure of his biological process.

## Figures and Tables

**Table 1.** Characteristics of wastewater and activated sludge used.

**Parameters**	**Concentrations**
***Wastewater***	
COD, mg·L^−1^	220 ± 105
TKN, mg·L^−1^	38.9 ± 7.5
NH_4_^+^-N, mg·L^−1^	24.5 ± 2.9
NO_3_^−^-N, mg·L^−1^	1.0 ± 0.3
Alkalinity, mg CaCO_3_·L^−1^	180 ± 10
***Activated sludge***	
MLVSS, mg·L^−1^	2,100 ± 150

**Table 2.** Concentrations of toxicants applied in this study.

**Toxic chemicals**	**Concentrations in reactor (mg·L^−1^)**				**Published inhibitory concentrations (mg·L^−1^)**
Hg^2+^	0	1.25	2.5	5.0	5 for activated sludge [32]
ATU	0	0.25	0.5	0.75	0.2–5.5 for activated sludge [33]
CHCl_3_	0	0.25	0.75	1.25	500 for activated sludge [34]
					0.48 for *Nitrosomonas sp.*[35]

**Table 3.** Percent nitrification inhibition and percent respiration inhibition calculated for toxicants of different concentrations.

**Toxic chemicals**	**Concentration, mg·L^−1^**	**Percent inhibition based on AV on pH profile, %***^a^*	**Percent inhibition based on OUR profile, %***^b^*
Hg^2+^	1.25	7.1 (1.3)	10.0 (5.7)
	2.5	33.3 (8.7)	34.6 (12.6)
	5	91.7 (2.7)	61.4 (7.7)
ATU	0.25	23.1 (4.3)	8.0 (0.2)
	0.5	72.9 (3.0)	34.3 (9.0)
	0.75	94.2 (6.5)	57.3 (24.3)
CHCl_3_	0.25	46.8 (8.1)	26.0 (7.6)
	0.75	54.0 (2.1)	34.1 (9.4)
	1.25	62.4 (8.9)	35.8 (5.1)

^a^ Based on Equation (2);

^b^ Based on Equation (3); ( ): standard deviations.

**Table 4.** Summary of screening tests with different toxicants.

**Toxic chemicals**	**Concentration, mg·L^−1^**	**Based on OUR**	**Based on dpH/dt**	**Final signal***^a^*
Hg^2+^	1.25	S	S	S
	2.5	T	T	T
	5	T	T	T
ATU	0.25	S	T	T
	0.5	T	T	T
	0.75	T	T	T
CHCl_3_	0.25	S	T	T
	0.75	T	T	T
	1.25	T	T	T

S: “safe”; T: “toxic”;

^a^ If either the OUR or the dpH/dt profile indicates “toxic” for test wastewater, then the final signal was “toxic”.
